# Supplementary material for: Unified momentum model for rotor aerodynamics across operating regimes
Source: Nat Commun. 2024 Aug 21;15:6658. doi: 10.1038/s41467-024-50756-5 (PMC11339364; doi:10.1038/s41467-024-50756-5)
Supplement: Supplementary file 1 — Supplementary Information [file 41467_2024_50756_MOESM1_ESM.pdf]

Supplementary Information for: Unified Momentum Model for Rotor Aerodynamics Across Operating Regimes

Jaime Liew,[1] Kirby S. Heck,[1] Michael F. Howland[1*]

[1]Civil and Environmental Engineering, Massachusetts Institute of Technology,

*To whom correspondence should be addressed; E-mail: mhowland@mit.edu.

Supplementary Information

This PDF file includes:

Supplementary Materials and Methods

Supplementary Text

Supplementary Figs. 1 to 14

SI references

Supplementary Materials and Methods

Unified momentum model

As described in **Methods**, the final form of the equations is:

$$a_n = 1 - \sqrt{\frac{u_\infty^2 - u_4^2 - v_4^2}{C_T' \cos^2(\gamma) u_\infty^2} - \frac{(p_4 - p_1)}{\frac{1}{2}\rho C_T' \cos^2(\gamma) u_\infty^2}} \tag{1}$$

$$u_4 = -\frac{1}{4} C_T'(1 - a_n)\cos^2(\gamma) u_\infty + \frac{u_\infty}{2} + \frac{1}{2}\sqrt{\left(\frac{1}{2} C_T'(1 - a_n)\cos^2(\gamma) u_\infty - u_\infty\right)^2 - \frac{4(p_4 - p_1)}{\rho}} \tag{2}$$

$$v_4 = -\frac{1}{4} C_T'(1 - a_n)^2 \sin(\gamma)\cos^2(\gamma) u_\infty \tag{3}$$

$$\frac{x_0}{D} = \frac{\cos(\gamma)}{2\beta} \frac{u_\infty + u_4}{|u_\infty - u_4|}\sqrt{\frac{(1 - a_n)\cos(\gamma) u_\infty}{u_\infty + u_4}} \tag{4}$$

$$p_4 - p_1 = -\frac{1}{2\pi}\rho C_T'(1 - a_n)^2 \cos^2(\gamma) u_\infty^2 \arctan\left[\frac{1}{2}\frac{D}{x_0}\right] + p^{NL}(C_T', \gamma, a_n, x_0), \tag{5}$$

where the freestream incident wind speed is u_∞, the fluid density is ρ, the actuator disk diameter is D, and the wake spreading rate is $\beta = 0.1403$ as outlined in **Near-wake length and model parameter analysis**. The pressure equation (Eq. (5)) contains two terms for the pressure contribution of the outlet wake pressure. The first term is the pressure contribution from the actuator disk forcing, and the second term (p^{NL}) is a nonlinear term that results from the advection. The origin of both pressure terms is discussed in detail in the **Model derivation**.

If C_T is used as the input thrust coefficient variable instead of C_T', a sixth equation is included in the set of equations above:

$$C_T' = \frac{C_T}{(1 - a_n)^2 \cos^2(\gamma)}, \tag{6}$$

where the thrust coefficient is defined as $C_T = 2\|\vec{F}_T\|/(\rho A_d u_\infty^2)$, where $\|\vec{F}_T\|$ is the magnitude of the thrust force and $A_d = \pi D^2/4$ is the rotor area.

Model derivation

In this section, we provide the derivation of each of the five analytical model equations using control volume analysis around the actuator disk.

Thrust force Following Heck *et al.* (*1*), the thrust force for the actuator disk is defined as

$$\vec{F}_T = -\frac{1}{2}\rho C_T' A_d (\vec{u}_d \cdot \hat{n})^2 \hat{n}. \tag{7}$$

The rotor-normal, rotor-averaged induction is defined as

$$a_n = 1 - \frac{\vec{u}_d \cdot \hat{n}}{u_\infty \cos(\gamma)}, \tag{8}$$

which is the generalization of the standard (axial) induction factor in classical one-dimensional momentum modeling (*2*). Therefore, the thrust force can be written as a function of the rotor-normal induction factor

$$\vec{F}_T = -\frac{1}{2}\rho C_T' A_d (1-a_n)^2 \cos^2(\gamma) u_\infty^2 \left[\cos(\gamma)\hat{\imath} + \sin(\gamma)\hat{\jmath}\right]. \tag{9}$$

The power for the actuator disk is computed as $P = -\vec{F}_T \cdot \vec{u}_d$.

Bernoulli equation This Bernoulli analysis is similar to classical one-dimensional momentum modeling, and the yawed porous actuator disk derivation of Heck *et al.* (*1*), but we do not assume that $p_4 = p_1$. The velocity is continuous over the porous actuator disk such that $\vec{u}_2 = \vec{u}_3$. The pressure just upwind of the porous disk is

$$p_2 = p_1 + \frac{1}{2}\rho \left(\|\vec{u}_1\|^2 - \|\vec{u}_2\|^2\right), \tag{10}$$

and the pressure just downwind of the porous disk is

$$p_3 = p_4 + \frac{1}{2}\rho \left(\|\vec{u}_4\|^2 - \|\vec{u}_2\|^2\right), \tag{11}$$

where in general $\|\vec{u}_4\|$ has two components, u_4 and v_4, given an arbitrary yaw misalignment angle γ. Combining the two pressure equations, yields

$$p_4 - p_1 = \frac{1}{2}\rho\left(\|\vec{u}_1\|^2 - \|\vec{u}_4\|^2\right) + \frac{\|\vec{F}_T\|}{A_d}, \tag{12}$$

where $\|\vec{F}_T\|/A_d = p_3 - p_2 = -\frac{1}{2}\rho C_T'(1-a_n)^2\cos^2(\gamma)u_\infty^2$, and substituting for the definition of the wind turbine thrust force $\|\vec{F}_T\|$ yields

$$p_4 - p_1 = \frac{1}{2}\rho\left(\|\vec{u}_1\|^2 - \|\vec{u}_4\|^2\right) - \frac{1}{2}\rho C_T'(1-a_n)^2\cos^2(\gamma)u_\infty^2. \tag{13}$$

Finally, solving for the rotor-normal induction a_n gives the first equation to the Unified Momentum Model:

$$a_n = 1 - \sqrt{\frac{u_\infty^2 - u_4^2 - v_4^2}{C_T'\cos^2(\gamma)u_\infty^2} - \frac{(p_4 - p_1)}{\frac{1}{2}\rho C_T'\cos^2(\gamma)u_\infty^2}}. \tag{14}$$

Streamwise momentum balance Reynolds Transport Theorem for momentum is used in the control volume illustrated as the dashed lines in Figure 1 of the main text. Assuming steady-state flow such that time-derivative terms vanish, Reynolds Transport Theorem for linear momentum in the streamwise direction states

$$\left[\frac{\mathrm{d}\vec{P}}{\mathrm{d}t}\right]_{CM} \cdot \hat{\imath} = \sum \vec{F}\cdot\hat{\imath} = \vec{F}_T\cdot\hat{\imath} + p_1 A_4 - p_4 A_4 = \int_{\mathrm{CS}} \rho u\left(\vec{u}_{\mathrm{rel}}\cdot \mathrm{d}\vec{A}\right), \tag{15}$$

where $\vec{P}$ is the linear momentum and CM denotes the control mass (*3, 4*). Simplifying this momentum conservation in the streamwise direction, assuming uniform flow within the streamtube, gives

$$\vec{F}_T\cdot\hat{\imath} = \rho u_4^2 A_4 - \rho u_\infty^2 A_4 + \dot{m}_{out}u_\infty + A_4(p_4 - p_1), \tag{16}$$

where $\dot{m}_{out} = \dot{m}_1 + \dot{m}_2$ is the mass flux out of the top and bottom of the control volume. The uniform flow assumption implies that the streamwise velocity and pressure on the downstream cylinder cross-sectional area is u_1 and p_1, respectively, except in the A_4 region, where they are

$\vec{u}_4$ and p_4, respectively. From mass conservation in the streamtube, we arrive at

$$\begin{aligned} u_4 A_4 &= (1-a_n)\cos(\gamma) A_d u_\infty \\ A_4 &= \frac{(1-a_n)\cos(\gamma) A_d u_\infty}{u_4}. \end{aligned} \tag{17}$$

From conservation of mass in the control volume, the mass flux out of the control volume is

$$\dot{m}_{out} = \rho A_4 (u_\infty - u_4). \tag{18}$$

Combining Equations (16) and (17), along with mass conservation in the control volume, we have

$$\vec{F}_T \cdot \hat{i} = \rho(1-a_n)\frac{u_\infty}{u_4}\cos(\gamma) A_d \left(u_4^2 - u_\infty u_4\right) + \frac{(1-a_n)\cos(\gamma) A_d u_\infty}{u_4}(p_4 - p_1). \tag{19}$$

The final equation for the outlet streamwise velocity u_4 is

$$u_4^2 + \left(\frac{1}{2} C_T'(1-a_n)\cos^2(\gamma) u_\infty - u_\infty\right) u_4 + \frac{1}{\rho}(p_4 - p_1) = 0, \tag{20}$$

which can be solved using the quadratic formula yielding the second equation to the Unified Momentum Model:

$$u_4 = -\frac{1}{4} C_T'(1-a_n)\cos^2(\gamma) u_\infty + \frac{u_\infty}{2} + \frac{1}{2}\sqrt{\left(\frac{1}{2} C_T'(1-a_n)\cos^2(\gamma) u_\infty - u_\infty\right)^2 - \frac{4(p_4 - p_1)}{\rho}}. \tag{21}$$

Lifting line model for the lateral wake velocity Following Shapiro *et al.* (*5*) and Heck *et al.* (*1*), Prandtl lifting line theory (*6*) is used to model the lateral velocity v_4 in the wake of a misaligned actuator disk. The model uses the downwash associated with an elliptic distribution of transverse (lateral) lift as the lateral velocity downwind of a yaw misaligned actuator disk. An elliptic lift distribution provides a constant downwash (lateral velocity) (*5, 6*). The circulation at the disk hub-height ($z = 0$), is defined as $\Gamma_0 = -2L/(\rho \pi R u_\infty)$, where $L = -\vec{F}_T \cdot \hat{j}$ is the

total lift force and R is the disk radius. From Milne-Thomson (1973) (*6*), the lateral velocity disturbance is therefore

$$\delta v_0 = v_\infty - v_4 = \frac{-\Gamma_0}{4R} = \frac{-\vec{F}_T \cdot \hat{j}}{2\rho u_\infty A_d} = \frac{1}{4} C_T' u_\infty \sin(\gamma)\cos^2(\gamma)\left(1 - a_n(\gamma)\right)^2. \qquad (22)$$

Therefore, the third equation of the model describing the lateral wake velocity is

$$v_4 = -\frac{1}{4} C_T' (1 - a_n)^2 \sin(\gamma)\cos^2(\gamma) u_\infty, \qquad (23)$$

since $v_\infty = 0$ as the freestream flow is only in the streamwise direction (see Figure 1, main document).

Near-wake length In determining the pressure drop over the control volume, $p_4 - p_1$, the streamwise location of the boundary of the control volume must be known. This near-wake length, denoted as x_0 and known as the potential core length in jet flow literature (*7*), is predicted following the approach proposed by Bastankhah & Porté-Agel (*8*), who generalized the shear layer model of Lee & Chu (*7*). The differential form of the shear layer width, s, is given by Lee & Chu (*7*) as

$$\left|\frac{u_s}{u_\infty}\right| \frac{\mathrm{d}s}{\mathrm{d}x} = \beta \left|\frac{u_e}{u_\infty}\right|, \qquad (24)$$

where $u_s = (u_\infty + u_4)/2$ is the characteristic shear layer velocity, $u_e = (u_\infty - u_4)/2$ is the characteristic relative velocity, and β is the wake or jet spread rate (*7*). The spreading rate β is an unknown parameter, for which we use $\beta = 0.1403$ in this study, which is within the standard range of this parameter within the literature, and which is further discussed in **Near-wake length and model parameter analysis**. The shear layer thickness at $x = x_0$ is given by Bastankhah & Porté-Agel (*8*) as $s/D = \frac{\cos(\gamma)}{2}\sqrt{\frac{u_2}{u_\infty + u_4}}$, where $u_2 = (1 - a_n)\cos(\gamma)u_\infty$ is the streamwise velocity at the disk. Integrating both sides yields the fourth equation of the model describing the near-wake length normalized by rotor diameter

$$\frac{x_0}{D} = \frac{\cos(\gamma)}{2\beta} \frac{u_\infty + u_4}{|u_\infty - u_4|} \sqrt{\frac{(1 - a_n)\cos(\gamma)u_\infty}{u_\infty + u_4}}. \qquad (25)$$

Outlet pressure modeling The final component needed to close the system of equations is the pressure difference across the control volume, $p_4 - p_1$. The pressure model proposed here is based on a solution to the steady, two-dimensional Euler equations with an actuator disk turbine body forcing proposed by Madsen (*9*), based on the analytical method of Von Kármán and Burgers (*10*). In the presented formulation, $p_4 - p_1$ can be decomposed into the sum of a linear pressure term p^L which contains contribution from rotor body forces, and a nonlinear pressure term p^{NL} containing contributions from flow advection. The linear term p^L accounts for the contribution from rotor body forces and has an analytical solution that can be solved in a straightforward manner. On the other hand, the nonlinear term p^{NL} includes contributions from flow advection and does not have a closed-form solution, requiring additional computational considerations. The derivation of both p^L and p^{NL} is described in this section, and the computational method for p^{NL} is described in **Nonlinear pressure field solution**.

The steady, two-dimensional incompressible Euler equations with constant density ρ are

$$u_j \frac{\partial u_i}{\partial x_j} = -\frac{1}{\rho}\frac{\partial p}{\partial x_i} + f_i, \tag{26}$$

where f_i represents the actuator disk body forcing in the ith direction. The conservation of mass is given by $\partial u_i/\partial x_i = 0$. The velocity field is decomposed into the background flow and actuator disk-induced components: $u = u_\infty + w_x$ and $v = w_y$, where there is no freestream flow in the lateral direction. Thus, conservation of mass can be reduced to $\partial w_x/\partial x + \partial w_y/\partial y = 0$. The resulting momentum equations for the induced velocities w_x and w_y with constant density ρ are (Madsen (*9*))

$$u_\infty \frac{\partial w_x}{\partial x} = -\frac{1}{\rho}\frac{\partial p}{\partial x} + f_x - \left(w_x \frac{\partial w_x}{\partial x} + w_y \frac{\partial w_x}{\partial y} \right) \tag{27}$$

$$u_\infty \frac{\partial w_y}{\partial x} = -\frac{1}{\rho}\frac{\partial p}{\partial y} + f_y - \left(w_x \frac{\partial w_y}{\partial x} + w_y \frac{\partial w_y}{\partial y} \right), \tag{28}$$

where f_x and f_y represent the actuator disk body forcing. In incompressible flows, the divergence-free velocity condition is often used to provide a prognostic equation for the pressure (*11*). Here, we follow this approach, as in Madsen (*9*), and the conservation of mass becomes

$$\frac{\partial^2 p}{\partial x^2} + \frac{\partial^2 p}{\partial y^2} = \rho \left(\frac{\partial f_x}{\partial x} + \frac{\partial f_y}{\partial y} \right) + \rho \left(\frac{\partial g_x}{\partial x} + \frac{\partial g_y}{\partial y} \right), \tag{29}$$

where the nonlinear force field due to advection is consolidated into the terms g_x and g_y:

$$g_x = -\left(w_x \frac{\partial w_x}{\partial x} + w_y \frac{\partial w_x}{\partial y} \right) \tag{30}$$

$$g_y = -\left(w_x \frac{\partial w_y}{\partial x} + w_y \frac{\partial w_y}{\partial y} \right). \tag{31}$$

The pressure distribution surrounding a nearly infinitesimally thin actuator line uniformly loaded in two-dimensional space, under the assumption of inviscid (Euler) flow, can be expressed as the sum of two components: $p(x, y) = p^L(x, y) + p^{NL}(x, y)$. These pressure contributions can be decomposed given the linearity of the Poisson equation (Eq. (29)), given as

$$\frac{\partial^2 p^L}{\partial x^2} + \frac{\partial^2 p^L}{\partial y^2} = \rho \left(\frac{\partial f_x}{\partial x} + \frac{\partial f_y}{\partial y} \right) \tag{32}$$

$$\frac{\partial^2 p^{NL}}{\partial x^2} + \frac{\partial^2 p^{NL}}{\partial y^2} = \rho \left(\frac{\partial g_x}{\partial x} + \frac{\partial g_y}{\partial y} \right). \tag{33}$$

Here, p^L represents the pressure contribution derived from the linearized Euler equations, where there is no advection effect, and p^{NL} signifies the nonlinear contribution arising from advection. The nonlinear pressure term is particularly important to include in high-thrust scenarios to capture the persistent pressure drop far downstream of the actuator disk (Figure S1). The streamwise and lateral velocity deviations from the mean, w_x and w_y are also decomposed into a linear and nonlinear component:

$$w_x = w_x^L + w_x^{NL} \tag{34}$$

$$w_y = w_y^L + w_y^{NL}. \tag{35}$$

When inserting these decompositions into the two-dimensional Euler equations (Eq. (27)), the momentum equations for the linear induced velocities are

$$u_\infty \frac{\partial w_x^L}{\partial x} = -\frac{1}{\rho}\frac{\partial p^L}{\partial x} + f_x \tag{36}$$

$$u_\infty \frac{\partial w_y^L}{\partial x} = -\frac{1}{\rho}\frac{\partial p^L}{\partial y} + f_y. \tag{37}$$

As established by Madsen (2023) (*9*), an analytical solution exists for p^L:

$$p^L(x,y) = -\frac{\Delta p}{2\pi}\left(\arctan\left(\frac{D+2y}{2x}\right) + \arctan\left(\frac{D-2y}{2x}\right)\right), \tag{38}$$

where $\Delta p = p_2 - p_3 = -\|\vec{F}_T\|/A_d = \frac{1}{2}\rho C_T'(1-a_n)^2\cos^2(\gamma)u_\infty^2$ is the pressure drop over the rotor, and x and y are normalized by the rotor diameter. This normalization approach differs from the one presented by Madsen (2023), who instead normalized by the rotor radius. Therefore, the centerline pressure at the near-wake length ($x = x_0, y = 0$), required in Eq. (5), can be expressed as:

$$p_4 - p_1 = p^L(x = x_0, y = 0) + p^{NL}(x = x_0, y = 0) \tag{39}$$

$$= -\frac{1}{2\pi}\rho C_T'(1-a_n)^2\cos^2(\gamma)u_\infty^2 \arctan\left(\frac{1}{2}\frac{D}{x_0}\right) + p^{NL}(x = x_0, y = 0) \tag{40}$$

The linear components w_x^L and w_y^L also have an analytical solution as described in (*9*)

$$w_x^L(x,y) = \frac{\Delta p}{2\pi\rho u_\infty}\left(\arctan\left(\frac{D+2y}{2x}\right) + \arctan\left(\frac{D-2y}{2x}\right)\right) - \underbrace{\Delta p/(\rho u_\infty)}_{\text{when behind rotor only}} \tag{41}$$

$$w_y^L(x,y) = \frac{\Delta p}{4\pi\rho u_\infty}\ln\left(\frac{4x^2 + (2y+D)^2}{4x^2 + (2y-D)^2}\right). \tag{42}$$

In the present coordinate system, the center of the actuator disk is at $x = 0$ and $y = 0$, so the last term in the equation for $w_x^L(x,y)$ (Eq. (41)) appears only for $x > 0$ and $|y| < D/2$.

The nonlinear pressure p^{NL} does not permit an analytical solution as the linear pressure p^L does, due to the presence of advection. The momentum equations for the nonlinear induced

velocities are

$$u_\infty \frac{\partial w_x^{NL}}{\partial x} = -\frac{1}{\rho}\frac{\partial p^{NL}}{\partial x} + g_x \tag{43}$$

$$u_\infty \frac{\partial w_y^{NL}}{\partial x} = -\frac{1}{\rho}\frac{\partial p^{NL}}{\partial y} + g_y. \tag{44}$$

The associated prognostic equation for the nonlinear pressure stems from the conservation of mass:

$$\frac{\partial^2 p^{NL}}{\partial x^2} + \frac{\partial^2 p^{NL}}{\partial y^2} = \rho \left(\frac{\partial g_x}{\partial x} + \frac{\partial g_y}{\partial y} \right). \tag{45}$$

Note that the nonlinear pressure contribution p^{NL} depends on the nonlinear terms g_x and g_y which depend on the full induced velocities w_x and w_y including their linear and nonlinear components. Therefore, Eq. (45) is the Poisson equation that must be solved for predictions of pressure. The pressure-Poisson equation, Eq. (45) is solved using a Green's function approach, yielding a solution of the form

$$p^{NL}(x,y) = \frac{\rho}{2\pi} \iint_{\mathbb{R}^2} \frac{g_x(x-x') + g_y(y-y')}{(x-x')^2 + (y-y')^2} dx' dy'. \tag{46}$$

To facilitate a numerical solution (see **Nonlinear pressure field solution**), this can be expressed as the sum of two convolutions:

$$p^{NL}(x,y) = \frac{\rho}{2\pi} \left(g_x \circledast \frac{x}{x^2+y^2} + g_y \circledast \frac{y}{x^2+y^2} \right) \tag{47}$$

where $\circledast$ is the two-dimensional convolution operator. Finally, the velocity components are updated by solving Equations (43) and (44), which can be rearranged as:

$$w_x^{NL}(x,y) = \frac{-p^{NL}}{\rho u_\infty} + \int_{-\infty}^{x} \frac{g_x}{u_\infty} dx' \tag{48}$$

$$w_y^{NL}(x,y) = \int_{-\infty}^{x} \left(-\frac{1}{\rho u_\infty} \frac{\partial p^{NL}}{\partial y} + \frac{g_y}{u_\infty} \right) dx'. \tag{49}$$

The required nonlinear pressure is evaluated at $p^{NL}(x = x_0, y = 0)$, similar to the linear pressure contribution. This yields the final form of $p_4 - p_1 = p^L(x = x_0, y = 0) + p^{NL}(x = x_0, y = 0)$.

Among the other variables in the Unified Momentum Model, p^{NL} is a function of C_T', γ, a_n, and x_0, and is therefore written as $p^{NL}(C_T', \gamma, a_n, x_0)$ in Eq. (5).

Due to the tight coupling between equations (43) - (49), the nonlinear pressure system is solved iteratively, as described in detail in **Model iterative solution**. As shown in Figure S1, the nonlinear pressure contribution was found to be negligible for low values of rotor thrust ($\Delta p/\rho u_\infty^2 \lessapprox 0.4$ or $C_T \lessapprox 0.8$), but was influential in the high-thrust states. This is particularly true further downstream ($x \gtrapprox D$) where the wake experiences a lingering pressure drop due to base suction. If the p^{NL} is ignored, the system of equations becomes fully analytical, but the pressure drop over the control volume is underestimated, causing under-predictions of u_4, C_P, and C_T as shown in Figure S6. By including the nonlinear pressure term, the Unified Momentum Model shows excellent agreement with LES in all operating regions.

Variation with C_T as input Although the above derivation is formulated using the modified thrust coefficient, C_T', it is common in blade-element methods to use C_T as an input instead. This can be achieved by adding an optional sixth equation to the above set of five which relates C_T and C_T' as described in (*1*):

$$C_T' = \frac{C_T}{(1 - a_n)^2 \cos^2(\gamma)}, \tag{50}$$

where the thrust coefficient is $C_T = 2\|\vec{F}_T\|/(\rho A_d u_\infty^2)$.

Model iterative solution

The presented unified equations do not permit a closed-form solution and must be solved iteratively. For a given thrust coefficient (either C_T' or C_T) and yaw angle γ, fixed point iteration provides an efficient approach, whereby Eqs. (1) - (5) are recast into residual equations, $\mathbf{r}(\mathbf{x})$. Starting from initial guess $\mathbf{x}^{(0)} = [a_n^{(0)}, u_4^{(0)}, v_4^{(0)}, x_0^{(0)}, (p_4 - p_1)^{(0)}]^T$ (with the addition of $C_T'^{(0)}$ if C_T is used as the input thrust coefficient variable), the iterations proceed according to:

$$\mathbf{x}^{(k+1)} = \mathbf{x}^{(k)} + (1-\omega)\mathbf{r}(\mathbf{x}^{(k)}) \tag{51}$$

until convergence when $||\mathbf{r}(\mathbf{x}^{(k)})||_\infty < \epsilon$ for a desired tolerance, ϵ. Here, ω is a relaxation parameter between 0 and 1 which improves numerical stability. $\omega = 0.25$ provides stable results with fast convergence (<20 iterations) when using C_T' as an input for the windmill and turbulent wake state, but requires about 80 iterations for strongly negative thrust values in the propeller state. When using C_T as an input, convergence slows ($\sim 10\times$ more iterations), but the final results remain the same.

The initial guess, $x^{(0)}$, should ensure convergence for all valid inputs. When using C_T' as an input, initial values from the limiting case of $|v_4| \ll u_4$ and $p_4 - p_1 = 0$ (as in Heck *et al.* (*1*)) provide stable results:

$$a_n^{(0)} = \frac{C_T' \cos^2(\gamma)}{4 + C_T' \cos^2(\gamma)} \tag{52}$$

$$\frac{u_4^{(0)}}{u_\infty} = \frac{4 - C_T' \cos^2(\gamma)}{4 + C_T' \cos^2(\gamma)} \tag{53}$$

$$\frac{v_4^{(0)}}{u_\infty} = \frac{4 C_T' \sin(\gamma) \cos^2(\gamma)}{(4 + C_T' \cos^2(\gamma))^2} \tag{54}$$

$$\frac{x_0^{(0)}}{D} \to \infty \tag{55}$$

$$(p_4 - p_1)^{(0)} = 0 \tag{56}$$

$$\tag{57}$$

where the initial guess for $x_0^{(0)}/D$ is large (≈ 100).

Nonlinear pressure field solution

The nonlinear pressure field described in Equations (43) - (49) remains the most complex part of the presented model, requiring an iterative solution to a 2D pressure field, from which the

centerline pressure behind the actuator disk can be extracted. This section describes how to arrive at the function $p^{NL}(C_T', \gamma, a_n, x_0)$ which is present in the last equation of the Unified Momentum Model, Equation (5).

Equations (43) - (49) are solved iteratively, starting from an initial guess of $w_x^{NL}(x, y) = w_y^{NL}(x, y) = 0$ and $p^{NL}(x, y) = 0$. The iteration process is as follows. First, the nonlinear forcing terms are computed using Equations (30) and (31) based on the sum of the linear and nonlinear velocity components in Equations (34) and (35). The derivatives can be solved numerically using finite differencing. The linear components, w_x^L and w_y^L have analytical forms shown in Equations (41) and (42), which are a function of $\Delta p = \rho C_T'(1 - a_n)^2 \cos^2(\gamma)$.

Next, the convolution solution to the pressure-Poisson equation, Equation (47) is solved. This can be quickly solved using numerical libraries which solve the convolution in Fourier space. Finally, the nonlinear induced velocities w_x^{NL} and w_y^{NL} in Eq. (49) are solved numerically using trapezoidal rule integration and finite differencing. These three steps are iterated until the centerline pressure field is adequately resolved. If computational speed is of paramount importance in model evaluation, the resulting nonlinear centerline pressure can then be tabulated as a function of $\Delta p/(\rho u_\infty^2) \in [-1, 1]$ and $x \in [0, \sim 20D]$ (Figure S1b), and used as a lookup table during runtime evaluation of the Unified Momentum Model equations (Eq. (1)-Eq. (5)).

When numerically solving for the pressure field induced by a high thrust actuator disk using the inviscid, steady-state Euler equations (Eqs. (27) and (28)), divergence of the iterative solver was encountered even with refined discretization. At low C_T, the divergence was not present. For higher C_T, divergence typically appears between 5 and 15 iterations, but this depends on the input thrust coefficient C_T. This indicates an instability inherent to the mathematical modeling form in the pressure Poisson equation (Eq (29)) that stems from the two-dimensional Euler equations with a sharp actuator disk forcing. For positive C_T, the nonlinear contribution to the wake pressure is negative. To approximate the nonlinear pressure p^{NL} before divergence, we

monitor the minimum pressure values at each center-line ($y = 0$) location throughout iterations of a range of relaxations. For positive values of C_T, the instability in the pressure model tends to drive pressure values in the wake towards positive infinity. Thus, recording the minimum pressure at each center-line location offers an approximate upper bound for the magnitude of the nonlinear pressure drop. While not ensuring an exact representation of the true pressure drop, constructing an upper-bound envelope provides a heuristic for capturing dominant pressure profile characteristics before the onset of instability.

While p^{NL} requires more complex considerations regarding its calculation, the provided upper bound value is justified when considering the *a priori* analysis performed in this study, which confirms the model form of the Unified Momentum Model, and the *a posteriori* analysis which demonstrates the predictive accuracy of the model as a whole. The presented methodology for estimating p^{NL} is therefore considered a fair compromise between accuracy and computational speed and simplicity. While other methods, such as Reynolds-Averaged Navier–Stokes, convergence stabilization methods (*12*), or a three-dimensional extension of the formulation, could potentially provide a better estimate of p^{NL}, they are not included since the presented upper bound formulation adequately predicts the key values in the Unified Momentum Model without the need of added complexity. Nevertheless, the accuracy of this approximation compared to the actual pressure field remains subject to further analysis, and additional mathematical investigation into the inherent modeling instability is warranted.

Near-wake length and model parameter analysis

The near-wake length x_0 in Equation (25) is derived using the Lagrangian shear layer model of Lee & Chu (*7, 13*) (see **Model derivation** for full description). The resulting near-wake length depends on an unknown parameter β that describes the wake (jet) spreading rate. Albertson *et al.* (1950) (*14*) found the spreading rate parameter as $\beta = 0.154$. Other values that have been

reported based on empirical data are $\beta = 0.116$ (*15*), $\beta = 0.119$ (*16*), and $\beta = 0.13$ (*17*). On the other hand, Bastankhah *et al.* (2016) (*8*) used $\beta = 0.154/2 = 0.077$.

In Figure S2, we evaluate the dependence of the near-wake length x_0 on the local thrust coefficient C_T'. In the near-wake region, the streamwise velocity monotonically decreases until it reaches its minimum value at x_0 (*5, 8*), after which the potential core vanishes, the wake velocity deficit transitions to a self-similar Gaussian profile (*7*), and the wake recovers (*5, 8, 18*). Therefore, the near-wake length is estimated from LES as the x location in the wake where the streamtube averaged streamwise velocity gradient reaches its minimum value and transitions to $\partial \overline{u}/\partial x > 0$, where $\overline{u}$ is the streamtube averaged streamwise velocity. The model equation for the near-wake length is in Eq. (25). The unknown parameter β in Eq. (25) is estimated by minimizing the mean square error between the model (with the nonlinear pressure neglected $p^{NL} = 0$) and the LES measured x_0, shown in Figure S2. A second estimate of β is produced by minimizing the mean square error between the model and the LES measurement of x_0 for the LES data where C_T is an input, in addition to the data where C_T' is an input (Figure S2). To emphasize the importance of the pressure deficit for high-thrust rotors, and given arbitrarily selected thrust coefficient values simulated here, the mean square error is calculated for $C_T' > 4$, and for $a > 0.5$ for the LES data where C_T is the thrust coefficient input. The resulting values of β are 0.1269 and 0.1537 for the cases where C_T' and C_T are input variables to the LES, respectively. Both values of β found through comparison of the model x_0 to the LES measured x_0 location are within the standard range of β values in the literature discussed previously (0.077-0.154).

The value of β used in this study is the average of the two values identified through the LES comparison, $\beta = (\beta_{\text{high}} + \beta_{\text{low}})/2 = (0.1269 + 0.1537)/2 = 0.1403$. To account for inherent uncertainty in the value of the unknown parameter β, we consider $\pm 10\%$ uncertainty about the mean value throughout this study, which is half of the average deviation $2(\beta_{\text{high}} - \beta_{\text{low}})/(\beta_{\text{high}} + \beta_{\text{low}}) = 19.1\%$. We note that the near-wake length x_0 accounts for arbitrary yaw misalignment

Table S1: Sensitivity of model outputs, $S = (dy/dx)(x/y)$ to variations in pressure drop and shear layer growth rate, β. Derivative is calculated at $\beta = 0.1403$. ($C_T' = 2$, $\gamma = 0^o$).

**Input**	**Output**				
	a_n	u_4	$p_4 - p_1$	C_P	C_T
$p_4 - p_1$	-0.0101	0.1290	-	0.0150	0.0100
β	-0.0104	0.1337	1.0423	0.0154	0.0103

values, and therefore the parameter has only been optimized for $\gamma = 0°$ in Figure S2. Using the value of $\beta = 0.1403$ in the model form, we show out-of-sample predictions of the near-wake length x_0 in yaw-misaligned conditions in Figure S3. Values of x_0 are extracted from uniform inflow LES for comparison with the model. The variation in the near-wake length from LES is primarily driven by changes in the thrust coefficient C_T'. For the same rotor-normal induction factor a_n, the unified model predicts monotonically decreasing near-wake length x_0 as the yaw misalignment angle increases, as shown in Figure S3(a). These model predictions qualitatively match the near-wake length observed in LES. When considering different yaw misalignment angles for the same thrust coefficient C_T', as shown in Figure S3(b), the variation in x_0 predicted by the model is compressed due to the nonlinear mapping between C_T' and a_n for a given yaw misalignment angle.

Sensitivity The Unified Momentum Model provides a physics-based closure for outlet pressure. To analyze model sensitivity, we perform a first-order study on including outlet pressure effects and the shear layer growth rate parameter β.

Sensitivity is assessed by taking the derivative of key output variables with respect to the parameter of interest. This linear analysis quantifies the influence of outlet pressure modeling and shear layer growth assumptions on model predictions.

This analysis shows streamwise outlet velocity is particularly sensitive to pressure variations, more so than other variables. This accounts for variations in the Steiros & Hultmark (2018)

(*19*) model's predictions of wake velocity and outlet pressure, despite maintaining favorable consistency with power coefficient and induction.

The pressure drop follows an inviscid model (see **Model derivation**) dependent on β. While influential on pressure and outlet velocity, β exhibits less sensitivity than pressure itself. Based on the sensitivities in Table S1, using the 20% margin of error in the presented estimate of β would lead to an approximate 20.8% margin of error in the pressure drop, but only a 2.7% margin of error in the outlet velocity, u_4, at fixed inputs of $C_T' = 2$ and $\gamma = 0^o$. Figs. S4 and S5 show selected sensitivities vs. C_T' and γ. The outlet velocity's β-sensitivity rises with C_T', while the pressure's decreases.

Supplementary Note

Comparison to source panel method modeling

A parallel modeling approach to the methodology presented here represents the porous disk with a distribution of equal magnitude sources in potential flow (*20–22*). However, these approaches have also in large part neglected the wake pressure deficit. Steiros & Hultmark (*19*) extended the source modeling approach by providing a more detailed representation of the wake, using momentum conservation and the Bernoulli equation, and by including a wake pressure term. Following the standard four-station analysis approach in one-dimensional momentum modeling (Figure 1, main document), the wake pressure deficit, $(p_4 - p_1)/\rho$, usually assumed to be zero in classical momentum modeling, can instead be left as a variable. To close the derived system of equations analytically, a wake factor $E = (u_2/u_\infty)/(2 - u_2/u_\infty)$ was introduced by Steiros & Hultmark (*19*), and the wake velocity was modeled as $u_4 = Eu_\infty$. The final analytical form of the model proposed by Steiros & Hultmark (*19*) is

$$C_T = \frac{4a(3-a)}{3(1+a)}, \qquad \frac{u_4}{u_\infty} = \frac{1-a}{1+a}, \qquad \frac{p_4 - p_1}{\rho} = -\frac{4}{3}u_\infty^2 \left(\frac{a}{a+1}\right)^2, \tag{58}$$

where C_T is the coefficient of thrust and u_4 is the near-wake velocity. Note that a is used to represent the induction factor, rather than a_n, because the one-dimensional model in Eq. (58) does not account for a potential misalignment between the porous disk and the incident wind. The model (Eq. (58)) exhibited excellent agreement compared to experimental measurements of the coefficient of drag (thrust) of porous plates immersed in a water tank (*19*). Further, this near-wake model was coupled to a far-wake model that also accounts for pressure recovery by Bempedelis & Steiros (*23*). While the thrust predictions of this model exhibit substantial improvements for high values of a compared to classical momentum modeling (*19*), it is notable that wake velocity model $u_4 = Eu_\infty = (1-a)/(1+a)$ differs from classical momentum modeling predictions $u_4 = 1 - 2a$ even for low induction values (below the heavily loaded limit). For example, at $a = 1/3$ the Betz limit value of induction that maximizes C_P in classical one-dimensional momentum modeling, the Steiros & Hultmark (*19*) model in Eq. (58) yields $u_4 = 1/2$, while momentum modeling yields $u_4 = 1/3$. As discussed previously, since near-wake models provide the wake initial conditions (i.e. u_4) to widely used turbulent far-wake models, it is important to investigate the predictive accuracy of u_4, in addition to C_T. The comparison to the Steiros & Hultmark (*19*) model is shown in Figure S6.

Empirical models for the high thrust coefficient regime A long-standing challenge has been reconciling discrepancies between classical momentum modeling predictions and empirical observations of highly loaded rotors operating in what is known as the *turbulent wake state*. In this state, the flow transitions from being attached and steady to fully separated and turbulent behind the rotor plane. Classical momentum modeling predicts decreasing thrust with increasing induction factor. Yet measurements show thrust continuing to rise, a phenomenon that Glauert addressed by introducing an empirical parabolic correction which overrides theory at high

inductions at $a > 0.4$ (*24*).

$$C_T = \begin{cases} 4a(1-a) & \text{for } a \leq 0.4 \\ 0.889 - \dfrac{0.203 - (a - 0.143)^2}{0.6427} & \text{for } a > 0.4 \end{cases} \tag{59}$$

Glauert is also associated with a second correction in the form of a cubic equation (*25*):

$$C_T = 4Fa(1 - f_g a) \tag{60}$$

where

$$f_g = \begin{cases} 1 & \text{for } a \leq 0.3 \\ \dfrac{1}{4}(5 - 3a) & \text{for } a > 0.3 \end{cases} \tag{61}$$

In subsequent decades, researchers have proposed various modifications to Glauert's empirical correction. Buhl later modified Glauert's parabola to remove discontinuities when incorporating tip loss corrections in blade element momentum models (*26*):

$$C_T = \begin{cases} 4Fa(1-a) & \text{for } a \leq 0.4 \\ \dfrac{8}{9}\left(4F - \dfrac{40}{9}\right)a + \left(\dfrac{50}{9} - 4F\right)a^2 & \text{for } a > 0.4 \end{cases} \tag{62}$$

Other empirical extensions were developed by researchers like Spera (*27*), Wilson (*28*), and Burton (*29*), employing linear relationships beyond a critical induction between $0.2 \leq a_c \leq 0.37$.

$$C_T = \begin{cases} 4Fa(1-a) & \text{for } a \leq a_c \\ 4F(a_c^2 + (1 - 2a_c)a) & \text{for } a > a_c \end{cases} \tag{63}$$

In contrast, Madsen *et al.* proposed a continuous cubic polynomial of a as a function of C_T to facilitate the typical mapping from C_T to a used in BEM methods (*30*):

$$a = k_1 C_T + k_2 C_T^2 + k^3 C_T^3 \tag{64}$$

where $k_1 = 0.2460$, $k_2 = 0.0586$, $k_3 = 0.0883$. The high-thrust corrections described in Equations (59), (60), (62), (63), (64), as well as the presented Unified Momentum Model are

presented in Figure S7. Numerous other variations exist in literature (see (*31*)), however, an analytical solution derived from first principles has remained elusive. Computational fluid dynamics (CFD) has enabled numerical modeling of stalled flow effects on wind turbines (*32*). But even with idealized uniform inflow, CFD simulations continue to exhibit the high-thrust phenomena, confirming the need to re-examine the underlying theory. This study provides an analytically derived Unified Momentum Model for rotor aerodynamics across the full operating envelope. By returning to first principles, the modifications to momentum theory are derived rather than empirically fitted. This physics-based analytical approach enables accurate modeling of rotors in high-thrust conditions without corrective factors while maintaining agreement with traditional momentum theory in windmill and propeller operation.

A unified blade element momentum model

In this section, we derive a new blade element momentum model using the Unified Momentum Model for the induction closure. The Unified Momentum Model replaces classical momentum theory as the induction closure. The Unified Momentum Model presented is extended to blade element momentum (BEM) methods by applying the streamtube assumptions to streamrings. This allows the formulation of a modified BEM approach without needing empirical Glauert corrections. The Unified Momentum Model provides additional insight including the near-wake characteristics, outlet velocities and pressure, and yaw misalignment losses that current empirical approaches do not accurately predict.

The modified BEM algorithm follows standard BEM procedures, except the induction factor closure uses the unified momentum system (Eqs. (1) - (5)). An additional equation (Eq. (6)) is incorporated relating the thrust coefficient in the unified equations to rotor-normal forces from blade element theory. This supplements the original five-equation system and closes the modified BEM method.

Input variables and geometry To model azimuthal variations due to yaw misalignment, a polar grid (μ, ψ) is defined over the turbine rotor. Here $\mu \in [0, 1] = r/R$ is the radial position normalized by rotor radius R, and ψ is the azimuthal angle. Turbine control operation set points are input as the yaw angle γ, blade pitch angle θ_p, and tip-speed ratio $\lambda = \Omega R/u_\infty$, where Ω is the angular velocity of the rotor. Rotor properties defined include:

- Airfoil lift and drag coefficients $C_l(\mu, \alpha)$ and $C_d(\mu, \alpha)$ as functions of μ and angle of attack α.
- Blade solidity $\sigma(\mu) = Bc(\mu)/2\pi R$, where B is number of blades and $c(\mu)$ is blade chord length as a function of μ.
- Blade twist angle $\theta_t(\mu)$ as a function of μ.

Defining these parameters enables modeling yaw misalignment effects and relating rotor forces to blade element momentum theory.

Blade forces The axial velocity (v_x) and the tangential velocity (v_t) for each blade sector are determined by considering the yaw misalignment and the azimuthal position, and they are expressed as follows:

$$v_x(\mu, \psi) = u_\infty(1 - a_n)\cos(\gamma) \tag{65}$$

$$v_t(\mu, \psi) = (1 + a')\lambda\mu - u_\infty(1 - a_n)\sin(\gamma)\cos(\psi) \tag{66}$$

The inflow angle ϕ and inflow velocity w are:

$$\phi(\mu, \psi) = \tan^{-1}(v_x/v_t) \tag{67}$$

$$w(\mu, \psi) = \sqrt{v_x^2 + v_t^2} \tag{68}$$

The angle of attack α, accounting for blade twist and pitch angle, is:

$$\alpha(\mu, \psi) = \phi(\mu, \psi) - \theta_t(\mu) - \theta_p \tag{69}$$

Finally, the rotor-normal and tangential force coefficients are:

$$C_n(\mu, \psi) = \cos(\phi) C_l(\mu, \alpha(\mu, \psi)) + \sin(\phi) C_d(\mu, \alpha(\mu, \psi)) \tag{70}$$

$$C_{tan}(\mu, \psi) = \sin(\phi) C_l(\mu, \alpha(\mu, \psi)) - \cos(\phi) C_d(\mu, \alpha(\mu, \psi)) \tag{71}$$

Thrust and torque balance As in standard BEM theory, the thrust and torque from momentum modeling are equated to blade element values. Here we present how both C_T and C_T' are determined. Forces are calculated per annulus by averaging over the azimuthal variations. The incremental thrust force, δT, over a radial segment, δr, is set equal between theories:

$$\delta T(\mu, \psi) = \frac{1}{2} \rho w(\mu, \psi)^2 B c(\mu) C_n(\mu, \psi) \delta r = \frac{1}{2} \rho C_T(\mu, \psi) u_\infty^2 2\pi r \delta r \tag{72}$$

Rearranging for C_T and azimuthally averaging gives the annulus thrust coefficient:

$$C_T(\mu) = \frac{\sigma(\mu)}{2\pi} \int_0^{2\pi} \frac{w(\mu, \psi)^2}{u_\infty^2} C_n(\mu, \psi) d\psi \tag{73}$$

$C_T'(\mu)$ can then be determined using

$$C_T'(\mu) = \frac{C_T(\mu)}{(1 - a(\mu))^2 \cos^2(\gamma)} \tag{74}$$

Using the presented Unified Momentum Model, C_T' relates to axial induction a_n. In this case $a_n(\mu) = g(C_T'(\mu)/F(\mu), \gamma)$ where $g(C_T', \gamma)$ is the converged solution of the five-equation Unified Momentum Model, Equations (1)-(5), which is solved iteratively as described in the **Model iterative solution** section, and $F(\mu)$ is the tip-loss correction for the blade element model, for which the following form is used in this investigation (*25*):

$$f_{\text{tip}} = \frac{B}{2} \frac{1 - \mu}{\mu \sin \phi} \tag{75}$$

$$f_{\text{root}} = \frac{B}{2} \frac{\mu - \mu_{\text{hub}}}{\mu \sin \phi} \tag{76}$$

$$F = \frac{2}{\pi} \arccos(\exp(-f_{\text{tip}})) \arccos(\exp(-f_{\text{root}})) \tag{77}$$

Similarly, equating the incremental torque between theories with tip-loss according to (*31*) and isolating $a'(\mu)$ gives:

$$\delta Q = \rho U_\infty F(\mu)(1 - a_n(\mu))\cos(\gamma)2\pi r 2a'(\mu)r^2\Omega\delta r = \frac{1}{2}\rho w(\mu,\psi)^2 Bc(\mu,\psi)rC_{tan}(\mu,\psi)\delta r \tag{78}$$

$$a'(\mu) = \frac{\sigma(\mu)}{8\pi F(\mu)\mu^2\lambda(1 - a_n(\mu))\cos(\gamma)}\int_0^{2\pi}\frac{w(\mu,\psi)^2}{u_\infty^2}C_{tan}(\mu,\psi)d\psi \tag{79}$$

The two equations which close the BEM loop are therefore:

$$a_n(\mu) = g(C_T'(\mu)/F(\mu),\gamma) \tag{80}$$

$$a'(\mu) = \frac{\sigma(\mu)}{8\pi F(\mu)\mu^2\lambda(1 - a_n(\mu))\cos(\gamma)}\int_0^{2\pi}\frac{w(\mu,\psi)^2}{u_\infty^2}C_{tan}(\mu,\psi)d\psi \tag{81}$$

This set of two induction factor equations can be solved independently at each rotor radial position using fixed point iteration with relaxation, as detailed in the **Model iterative solution** section. The Unified Momentum Model provides physics-based induction predictions, replacing traditional BEM implementations that rely on empirical factors. By solving these equations iteratively, the unified theory is incorporated into the blade element momentum algorithm, eliminating the need for empirical models in high-thrust or yawed operation.

Case study - IEA15MW reference turbine The new momentum theory presented in this work enables accurate modeling of wind turbine aerodynamics at high thrust and yaw misaligned conditions without needing empirical corrections. This is demonstrated by applying the described BEM method on the IEA 15MW Reference Wind Turbine (*33*).

Figure S8(a-f) shows the power coefficient calculated across a range of tip-speed ratios and blade pitch angles using different BEM approaches. Classical momentum theory without and with a high-thrust correction is adopted in aligned wind in Figures S8(a) and S8(b), and in yaw misaligned wind in Figures S8(d) and S8(e), respectively. The classical one-dimensional

momentum theory without a high-thrust correction fails to converge in regions where the thrust coefficient exceeds unity, rendering it undefined. Consequently, the global optimal operating point using this model lies on the convergence boundary, preventing the accurate determination of the optimal set point. By including an empirical high-thrust correction, the upper left quadrant of the surface can be realized, allowing a globally optimal set point to be retrieved, but it requires empiricism to achieve. Replacing the empirical high-thrust correction with the Unified Momentum Model (Figure S8(c)) enables the recovery of the complete contour without any empirical corrections.

Observing the C_P contour when the rotor is yaw misaligned (Figures S8(d-f)), the contour decreases in magnitude at all locations. The global optimal set points using the classical momentum theory indicate a decrease in the tip-speed ratio when the rotor is misaligned, corresponding to a decrease in thrust. Conversely, the Unified Momentum Model shows a decrease in pitch angle when the rotor is yaw-misaligned, indicating an increase in thrust. This increase in thrust aligns with literature suggesting that optimal control of wind turbines with yaw misalignment corresponds to an increase in thrust level (*1, 34*).

To evaluate the impact of yaw misalignment on rotor power output and rotor thrust predicted by the BEM implementation using the Unified Momentum Model, comparisons are made with blade-resolved Reynolds Averaged Navier Stokes simulations using the ExaWind stack (*35, 36*) for the IEA 15MW Reference Wind Turbine. The turbine was simulated at a fixed blade pitch angle of 0° and a tip-speed ratio of 9.0, with results shown in Figure S9. A baseline comparison was conducted using the classical BEM theory, which exhibited a cosine exponent close to 3 as incorrectly predicted by previous momentum theory implementations. The BEM model employing the Unified Momentum Model demonstrated both power and thrust reductions due to yaw misalignment, aligning more closely with the blade-resolved Reynolds-Averaged Navier–Stokes simulations. The Unified Momentum Model shows close to a threefold reduction in

power prediction error in yaw, with a 5.9% error at a 30° yaw angle compared to the BEM model using classical momentum theory which shows an error of 16.0 % at the same yaw angle.

Several additional comparisons are shown. First, the thrust coefficient $C_T(\gamma)$ is extracted from the blade-resolved simulation and fed directly into the Unified Momentum Model equations, which eliminates the need for the blade element closure. But this is not a predictive approach because the variation of $C_T(\gamma)$ with yaw γ must be known *a priori*. This comparison separates Unified Momentum Model errors from blade element model errors. Second, we use $C_T(\gamma)$ from the blade-resolved simulation to extract the power-yaw response for actuator disk model large eddy simulations if they followed the same thrust variation as a function of yaw ($C_T(\gamma)$). This comparison isolates errors associated with the prediction of actuator disk model aerodynamics alone and closely mirrors the errors shown in Figure 2 of the main text. Both of these results indicate that a sizable fraction of the 5.9% error at 30° yaw reported above from the novel BEM approach actually stems from the blade element modeling, rather than the Unified Momentum Modeling. This demonstrates further confidence in the Unified Momentum Model for actuator disk aerodynamics and suggests that future work should continue to improve on the blade element momentum formulation for advanced rotor modeling.

Overall, these examples demonstrate the capability of the unified momentum closure to enable physics-based modeling of wind turbine aerodynamics across the full operating envelope without empirical corrections. This will lead to more robust and generalizable aerodynamic models compared to traditional corrections based on fitting empirical data.

Streamtube analysis and budgets

In this section, we evaluate the quantities of interest within the streamtube enclosing the wake. We will perform *a priori* analysis of the assumptions within the proposed model. The control volume used in this analysis is the same as in the derivation of the Unified Momentum Model,

with a cross section larger than the streamtube containing the actuator disk, as shown in the dashed line in Figure 1 (main document). As in the Unified Momentum Model derivation, we consider four analysis stations and corresponding labels. We assume that the velocity is continuous across the (porous) actuator disk. The work done by the system $\dot{W}_{\text{system}}$ is the summation of the turbine work and the pressure work

$$-\dot{W}_{\text{system}} = -\dot{W}_{\text{turbine}} - \int_{CS} p\vec{u} \cdot \hat{n} \mathrm{d}A \tag{82}$$

$$= -\frac{1}{2}\rho A_d C_T'(1-a_n)^3 \cos^3(\gamma) u_\infty^3 + A_4 p_1 u_4 - A_4 p_4 u_4, \tag{83}$$

where CS is the control surface. From Reynolds Transport Theorem for kinetic energy, $e = \frac{1}{2}\|\vec{u}\|^2$, energy conservation in the control volume yields

$$-\dot{W}_{\text{system}} = \frac{\mathrm{d}}{\mathrm{d}t}\int_{CV} \rho e \mathrm{d}V + \int_{CS} \rho e \vec{u} \cdot \hat{n} \mathrm{d}A \tag{84}$$

$$= \frac{1}{2}\rho A_4 u_4 \|\vec{u_4}\|^2 - \frac{1}{2}\rho A_4 u_\infty^3 + \frac{1}{2} u_\infty^2 \dot{m}_{out} \tag{85}$$

$$= \frac{1}{2}\rho A_4 u_4 \|\vec{u_4}\|^2 - \frac{1}{2}\rho A_4 u_\infty^3 + \frac{1}{2}\rho u_\infty^2 A_4 (u_\infty - u_4) \tag{86}$$

$$= \frac{1}{2}\rho A_4 \left(u_4 \|\vec{u_4}\|^2 - u_4 u_\infty^2\right) \tag{87}$$

Here, we define p_4 relative to reference p_1, and we set $p_1 = 0$, yielding

$$-p_4 = \frac{1}{2}\rho C_T'(1-a_n)^2 \cos^2(\gamma) u_\infty^2 + \frac{\rho}{2}\|\vec{u_4}\|^2 - \frac{\rho}{2} u_\infty^2. \tag{88}$$

Note that Eq. (17) has been used to arrive at Eq. (88). Therefore, there is a pressure deficit in the wake region that is implied by energy conservation, as shown in Eq. (88). This result can also be derived from the Bernoulli equation which presents energy conservation in steady, irrotational flow. Consider the streamtube enclosing the wake. Energy conservation implied by the Bernoulli equation is considered in the streamtube

$$0 = \frac{1}{A}\int_A \Big(\underbrace{\frac{1}{2}\|\vec{u_4}\|^2}_{\text{Term I}} - \underbrace{\frac{1}{2}u_\infty^2}_{\text{Term II}} + \underbrace{\frac{p_4}{\rho}}_{\text{Term III}} \Big) \mathrm{d}A + \underbrace{\frac{1}{2}C_T'(1-a_n)^2 \cos^2(\gamma) u_\infty^2}_{\text{Term IV}}, \tag{89}$$

where A denotes the streamtube cross-sectional area, which depends on position x.

The streamtube averaged energy budget in Eq. (89) implies that from the energy contained in the incoming freestream flow (Term II), the portion of energy that is not extracted by the actuator disk (Term IV) is the summation of the energy associated with the outlet wake velocity $\|\vec{u_4}\|$ (Term I) and the outlet wake pressure p_4/ρ (Term III). Classical momentum modeling prescribes $p_4 = p_1$. For a given thrust (Term IV), enforcing that $p_4 = p_1$ can result in artificially low values of the outlet wake velocity magnitude $\|\vec{u_4}\|$. Alternatively, if there is a pressure deficit in the wake such that $p_4 < p_1$, higher values of the outlet wake velocity magnitude $\|\vec{u_4}\|$ can be achieved for a given thrust (Term IV). This explains why classical momentum modeling that assumes $p_4 = p_1$ predicts negative wake velocity for $a_n > 0.5$ ($C_T' > 4$), whereas the LES and Unified Momentum Modeling do not (see Figure S6).

The streamtube averaged streamwise velocity deficit, pressure deficit, and root-mean-square of the lateral velocity in the near-wake region are shown in Figure S10 for $0 < C_T' < 12$. From Figure S10(a), it is clear that classical one-dimensional momentum modeling with $p_4 = p_1$ increasingly over-predicts the magnitude of the wake velocity deficit at C_T' increases. Notably, wake velocity deficits greater than 1 imply negative (reverse) mean streamwise flow in the streamtube. The pressure in Figure S10(b) shows that increasing C_T' increases the thrust force (pressure drop at the disk multiplied by the disk area), but these marginal thrust increases diminish at higher values of C_T'. Further, there is a sustained pressure deficit in the wake region, and this wake pressure deficit is a monotonically increasing function of C_T'. Finally, the root-mean-square of the lateral velocity in Figure S10(c) demonstrates that there is an increase in lateral velocity perturbations at the disk, but that the lateral velocity is continuous across the disk, and that the root-mean-square of the lateral velocity is small in the inviscid region between $1 < x/D < 2$, where the streamwise velocity and pressure deficits are approximately constant before wake transition at approximately $x/D \gtrsim 2$ in these C_T' cases. Note, that while Steiros & Hultmark (*19*)

included the crosswise (spanwise) velocities at the disk in the Bernoulli analysis, the streamtube averaged root-mean-square of the spanwise velocity shown in Figure S10(c) indicates that the spanwise velocity is continuous across the disk (as is the streamwise velocity) and negligible in the inviscid near-wake region, and it, therefore, cancels from the Bernoulli analysis.

The streamtube averaged budget terms are shown in Figure S11 for $C_T' = 4$ and $\gamma = 0°$ and $\gamma = 40°$, in addition to $C_T' = 12$ and $\gamma = 0°$. These results indicate that within this near-wake region with uniform zero-turbulence inflow, approximately $1 < x/D < 2$, it is reasonable to approximate the budget terms with constant values that do not depend on x. In addition, the results indicate that the pressure deficit term ($\mathrm{Term\ III}$) is not negligible at these high values of C_T', and that neglecting this term leads to a residual in the energy budget (dashed line). The residual is higher for $C_T' = 12$ than for $C_T' = 4$, indicating the increasing magnitude of the wake pressure deficit relative to the thrust force, and its increasing importance in the energy balance. Further, the residual is higher for $\gamma = 0°$ than for $\gamma = 40°$, indicating that the magnitude of the wake pressure deficit, relative to the thrust force, decreases with increasing magnitude of the yaw angle.

In addition to the streamtube averaged budget terms, model approximations of the budget are also shown, where each term is approximated by a single averaged value that is characteristic of the 'near-wake.' This is consistent with the uniform inflow and wake flow assumption used within both the derivation of classical momentum theory and the Unified Momentum Model. The model approximation residuals are shown with and without the pressure contribution, to demonstrate the influence of neglecting the wake pressure p_4 in energy conservation. Comparing Figure S11(a) and Figure S11(c) which compute the model residuals for $C_T' = 4$ and $C_T' = 12$, respectively, and yaw alignment $\gamma = 0°$, we can see the growing influence of neglecting the wake pressure in the model. Comparing Figure S11(a) and Figure S11(b) which compute the model residuals for $C_T' = 4$ and yaw alignment $\gamma = 0°$ and yaw misalignment $\gamma = 40°$, respectively, we

can see the influence of neglecting the wake pressure depending on yaw. For the yaw misaligned case of $\gamma = 40°$, the streamtube averaged budget residual is smallest when including the pressure contribution. For the model results, the model residuals with pressure neglected are small and positive, while when pressure is included the model residuals are larger in magnitude and negative. This difference between budget and model results is related to the averaging operation over the streamtube cross-sectional area, as the difference between the budget results and the model results are that the model replaces the individual budget terms with a single, averaged value. The higher model residual for yaw misaligned porous disks is related to the increasing three-dimensionality in the wake of a yaw misaligned turbine (*37*). Going to a three-dimensional representation, as in the full budget shown in Figure S11, improves accuracy, but would increase the complexity of the model form and challenge arriving at an analytical form.

An alternative utility of the energy budget in Eq. (89), is that provided with estimates of the outlet velocity $\|\vec{u_4}\|$ and the rotor-normal induction a_n, the energy budget can be used to predict p_4, as shown in Eq. (88). In Figure S6(d), we compare predictions of the wake pressure deficit p_4, computed using Eq. (89) using the values of $\|\vec{u_4}\|$ (measured as described above) and a_n measured from LES, to the p_4 values measured from LES across the range of induction factors a_n. As shown in Figure S6(d), there is good agreement between the measured p_4 and the p_4 implied from energy conservation (Eq. (88)), which validates the fidelity of the energy conservation model form. As the induction factor a_n approaches a value of one, there is an increasing discrepancy between the LES measurements of p_4 and the p_4 that is implied by energy conservation (Eq. (88)), which is related to the reduced accuracy of the Bernoulli equation closer to $a_n = 1$. In addition, as a reference, we also show results from the predictive wake pressure deficit model from Steiros & Hultmark (*19*) from Eq. (58). While the predictive model from Steiros & Hultmark (*19*) in Eq. (58) captures the proper order of magnitude and the qualitative trends, such as the monotonic increase in wake pressure deficit as a function of increasing C_T'

(increasing induction factor a_n), the wake pressure deficit is consistently over-predicted until $a_n \approx 1$, where it underpredicts p_4 (not shown). In summary, the results indicate that the form of the model in Eq. (88) based on energy conservation in the near-wake is appropriate, with a reduction in the accuracy of the Bernoulli equation for very high induction values near $a_n = 1$.

Comparison between different actuator disk model regularization methods in LES

As discussed in the LES numerical setup in **Methods** of the main text, the ADM implemented in LES depends on the filter width Δ that dictates how the thrust force is distributed in the computational domain. In the limit of $\Delta \to 0$, the ADM trends towards an infinitesimally thin disk (consistent with actuator disk theory). However, a very small filter width Δ can lead to numerical instabilities, especially in the wake (*1, 38*). In this section, we compare four different numerical implementations of the ADM, considering both input of C_T' and C_T, and considering a relatively large filter width $\Delta/D = 0.1638 = (3/2)\sqrt{\Delta x^2 + \Delta y^2 + \Delta z^2}$ where the correction factor M derived by Shapiro *et al.* (2019) (*38*) is used, and a relatively small filter width $\Delta/D = 0.0325 = (0.29)\sqrt{\Delta x^2 + \Delta y^2 + \Delta z^2}$ where the correction factor is not used ($M = 0$). Finally, we also include LES results produced by Martinez *et al.* (2022) (*39*) (NREL), which did not use the Shapiro *et al.* (2019) (*38*) correction factor, and C_T was the input. In Figure S12, we compare the disk quantities (C_T and C_P) predicted by the large eddy simulations of an actuator disk model with different numerical implementations (regularization properties), depending on the induction factor a_n. In Figure S13, we show C_T and C_P depending on C_T'.

Overall, the qualitative results are similar, with small quantitative differences between the LES cases. The low filter width case with the correction factor turned off $M = 0$ tends to overpredict the thrust and power production (see Figure S13), as discussed extensively in the literature (*1, 38, 40*), because of the underprediction of the induction incurred by distributing the force beyond an infinitesimally thin disk with radius R. Also, the low filter width case results

in more numerical instabilities and noise in the measurements, as is evident with increasing induction a_n. However, these results demonstrate that the qualitative conclusions of this study are not affected by the choice of numerical setup for the ADM.

Unsteadiness in LES at high thrust

As the thrust coefficient, and correspondingly the thrust force, are increased, instabilities are triggered in the LES simulations, as documented by Sørensen *et al.* (1998) (*32*). This unsteadiness is related to the vortex ring and propeller brake states in the classical regime description of Stoddard (1978) (*41*). In their computational fluid dynamics simulations, Sørensen *et al.* (1998) (*32*) found that their simulations became unsteady at $C_T = 1.1$. The time series of the disk velocities u_2 for the different thrust coefficient C_T values simulated here are shown in Figure S14. For clarity of presentation in the main document, we present only results with a sufficiently small standard deviation ($\sqrt{\overline{u_2'^2}} < 0.15u_\infty$), such that the dynamics are well described by a time-averaged quantity (such as a_n, C_T, and C_P). After the initial startup transient in the LES, values below approximately $C_T \approx 1.3$ exhibit very small variance. For example, at $C_T = 1.3$, $\sqrt{\overline{u_2'^2}}/u_\infty = 1\%$. The transition to unsteadiness likely occurs at a higher C_T value in our LES, compared to the simulations of Sørensen *et al.* (1998) (*32*), because of the refined ADM regularization methodology of Shapiro *et al.* (2019) (*38*), and because our simulations are LES at infinite Reynolds number with a subgrid-scale closure (*42*), rather than computational fluid dynamics at a finite Reynolds number in Sørensen *et al.* (1998) (*32*).

Supplementary Figures

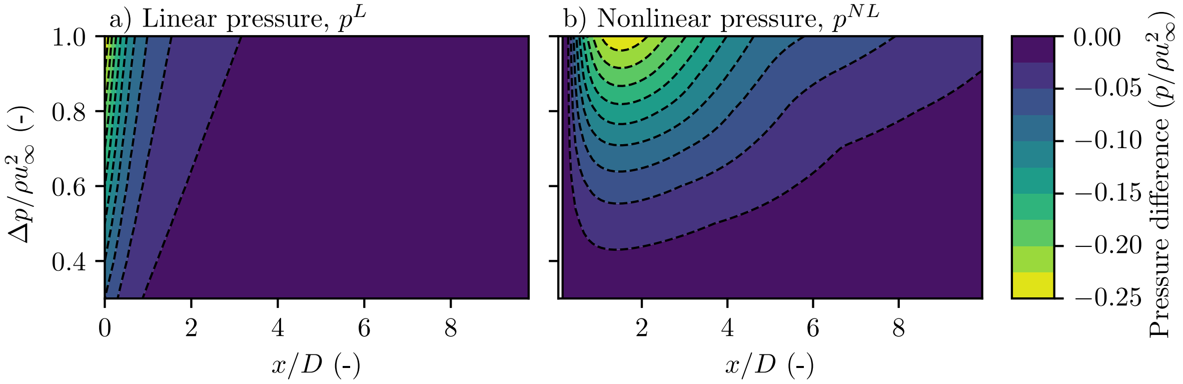

Figure S1: Centerline pressure behind the actuator disk as a function of downstream distance, x/D, and disk pressure drop, $\Delta p = p_2 - p_3 = \rho C_T'(1-a)^2 \cos^2(\gamma) u_\infty^2$. Pressure is decomposed into (a) the linear and (b) the nonlinear contribution.

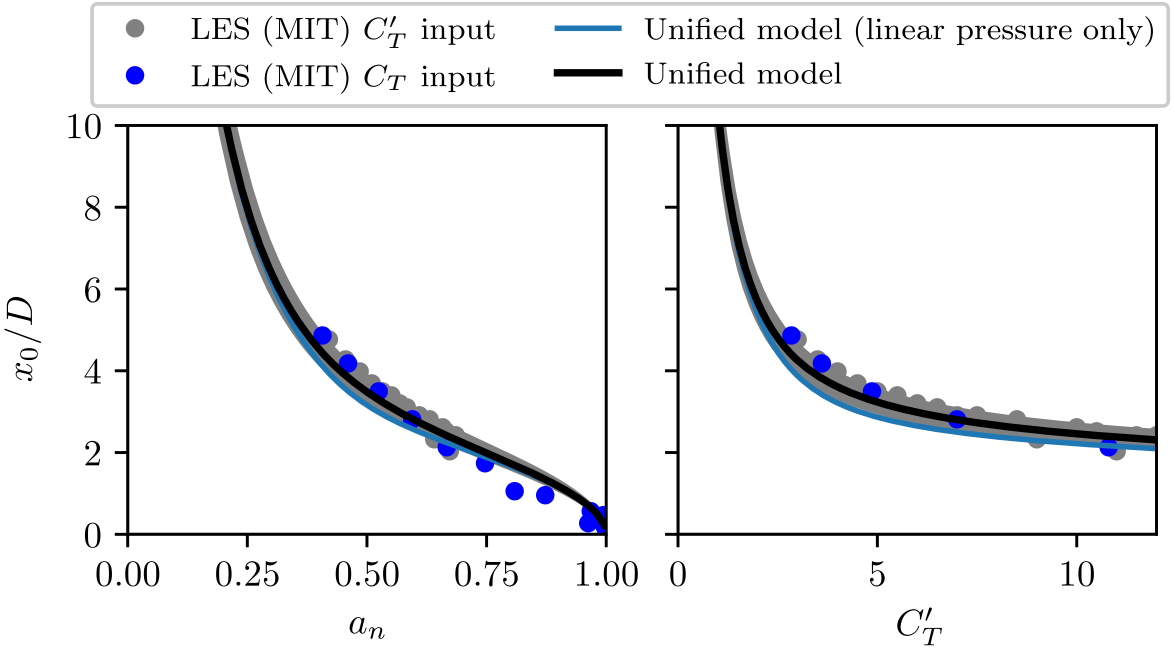

Figure S2: Near-wake length measured in LES and predicted by the near-wake length model shown in Eq. (25). The shaded region corresponds to $\pm 10\%$ uncertainty in β.

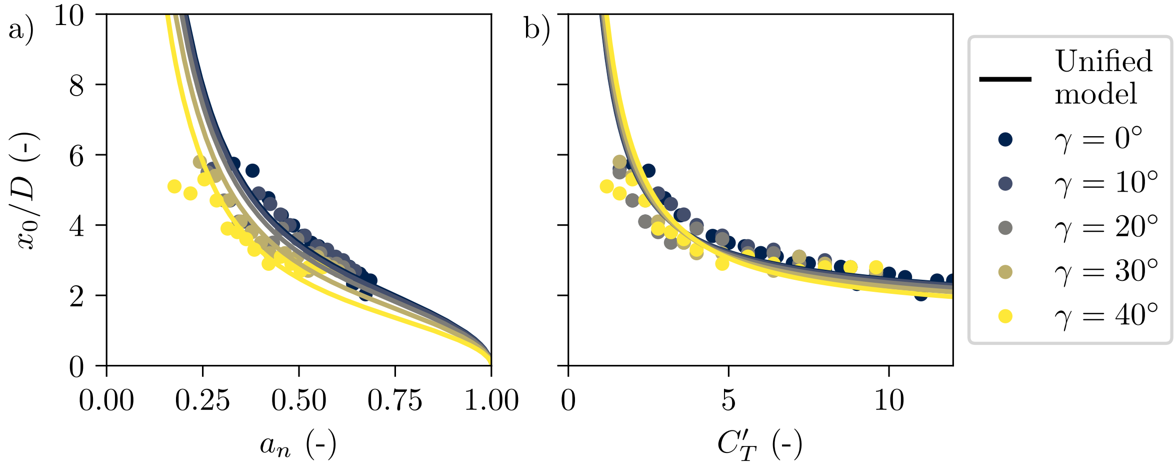

Figure S3: Near-wake length measured in LES and predicted by the near-wake length model (Eq. (25)) plotted against (a) induction factor and (b) thrust coefficient C_T' for different yaw misalignment angles.

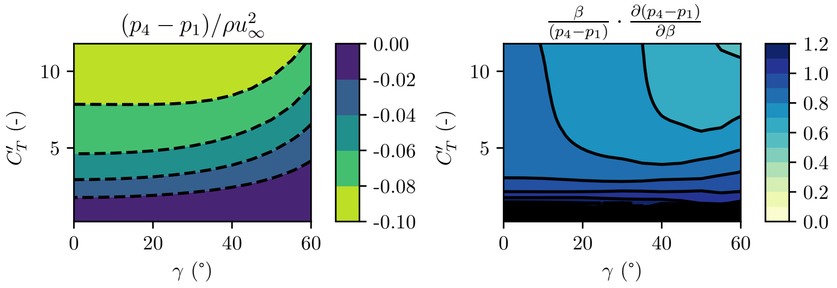

Figure S4: Pressure drop and its sensitivity with respect to shear layer growth rate parameter, β as a function of thrust coefficient, C_T' and yaw misalignment angle, γ.

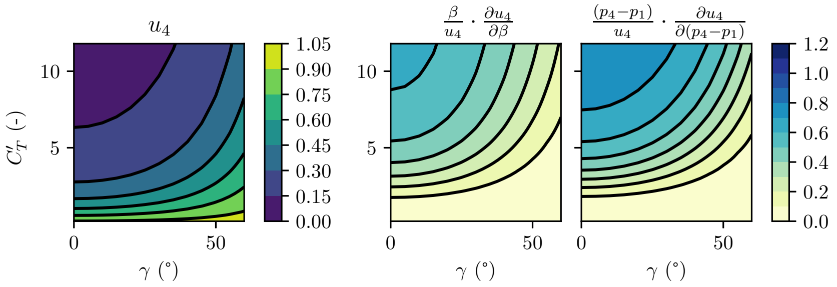

Figure S5: Streamwise outlet velocity, u_4 and its sensitivity with respect to pressure difference and β as a function of thrust coefficient, C_T' and yaw misalignment angle, γ.

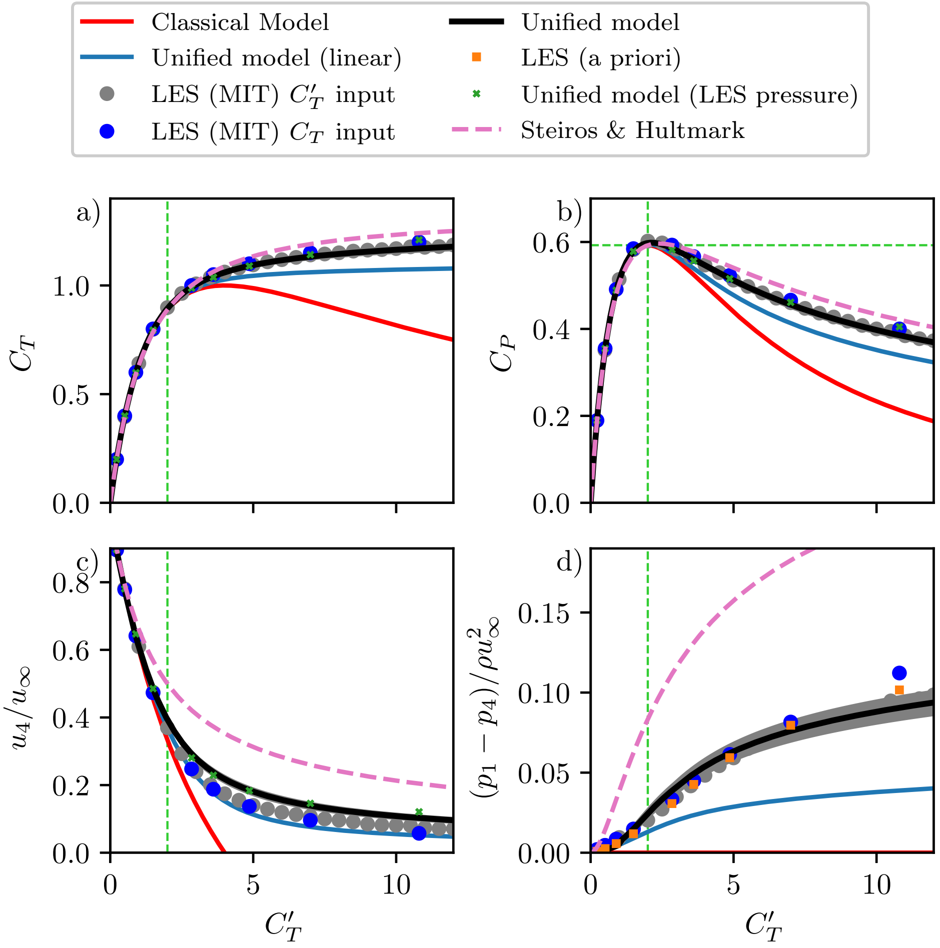

Figure S6: (a) Coefficient of thrust C_T, (b) Coefficient of power C_P, (c) streamwise wake velocity u_4, and (d) density-normalized wake pressure deficit $(p_1 - p_4)/\rho$ as a function of the local thrust coefficient C_T'. The variables estimated by the model equations proposed here (Eq. (1)) are shown. The shaded region corresponds to $\pm 10\%$ uncertainty in β. Results from LES, and classical one-dimensional momentum modeling are shown as a reference. The Betz limit of $C_T' = 2$ and $C_P = 16/27$ is also shown by the dashed green line. The model predictions from Steiros & Hultmark (*19*) (Eq. (58)) are also shown, as well as the Unified Momentum Model without the nonlinear pressure term.

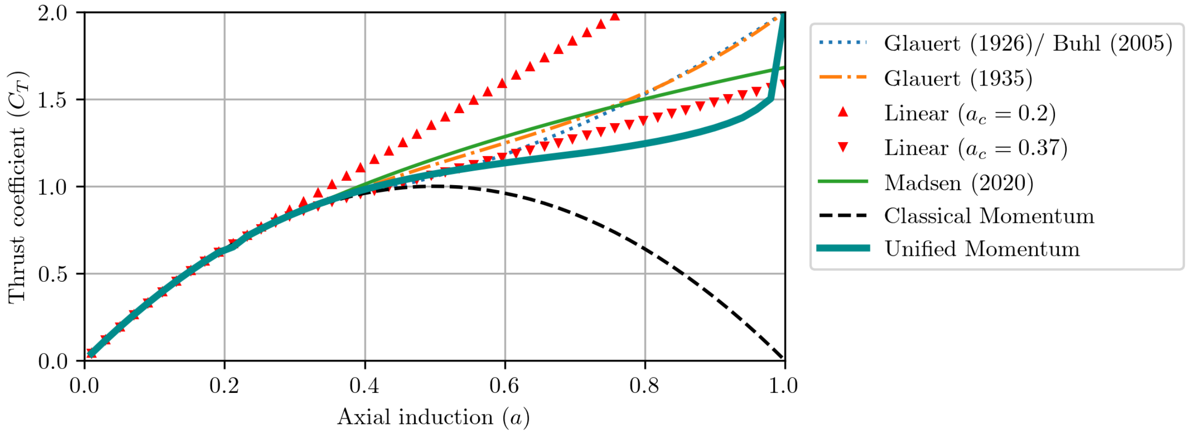

Figure S7: Relationship between thrust coefficient and axial induction as described by commonly used empirical models.

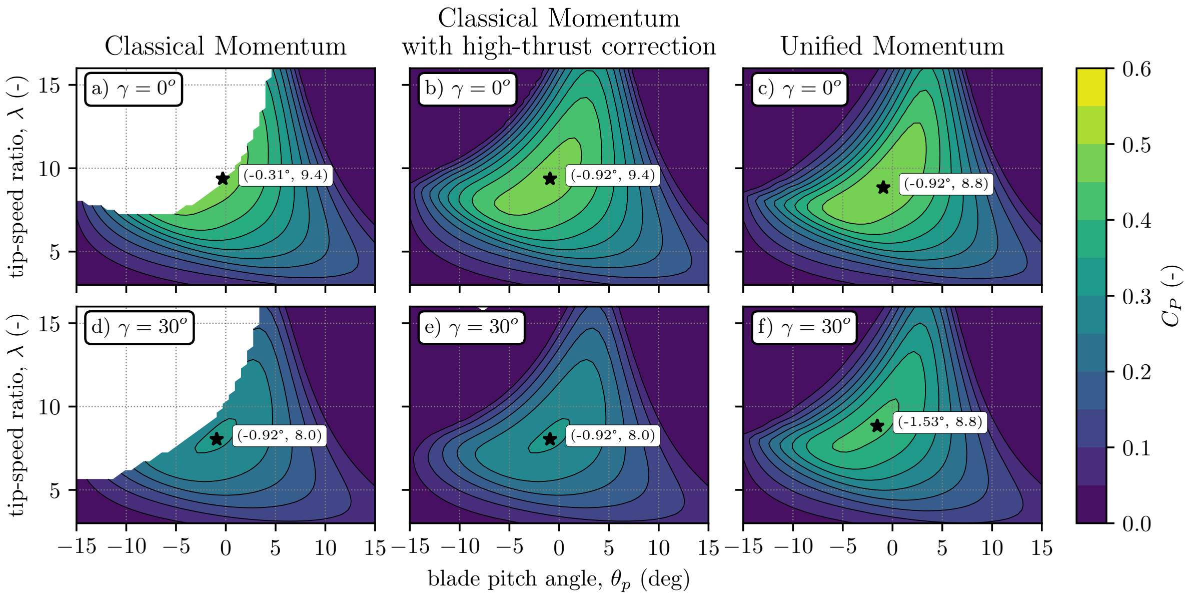

Figure S8: Contour plots showing the variation of power coefficient (C_P) with blade pitch angle (θ_p) and blade tip-speed ratio (λ) using different thrust-induction closures in a blade-element momentum implementation. Fully aligned (a, b, c) and yaw misaligned (d, e, f) conditions at $\gamma = 30^o$ are considered. Models include classical momentum without high-thrust correction (a, d), classical momentum with high-thrust correction (b, e), and the Unified Momentum Model (c, f), all incorporating Prandtl tip and root correction (*43*).

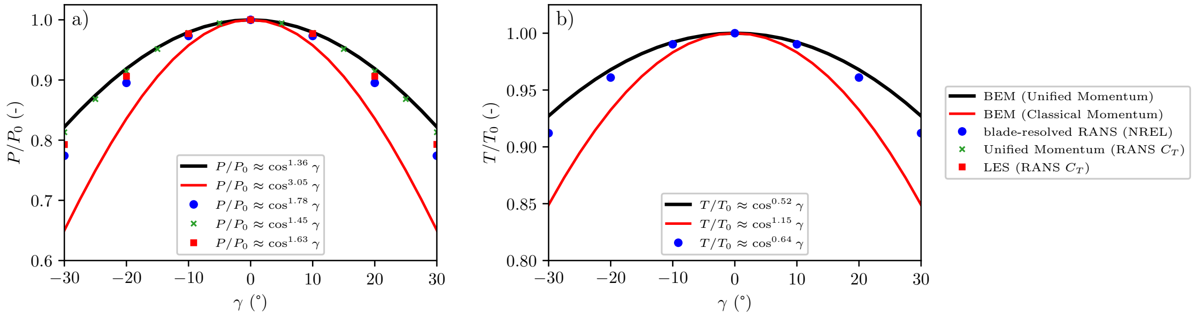

Figure S9: Comparison of (a) rotor power output and (b) rotor thrust at varying yaw angles using blade-element momentum models employing classical and unified momentum approaches, and blade-resolved Reynolds-averaged Navier–Stokes (RANS) simulations (*36*). In (a), additional data points show RANS thrust used as inputs to both the Unified Momentum Model and LES using PadéOps. Best-fit cosine exponents are noted in the legend for each model type.

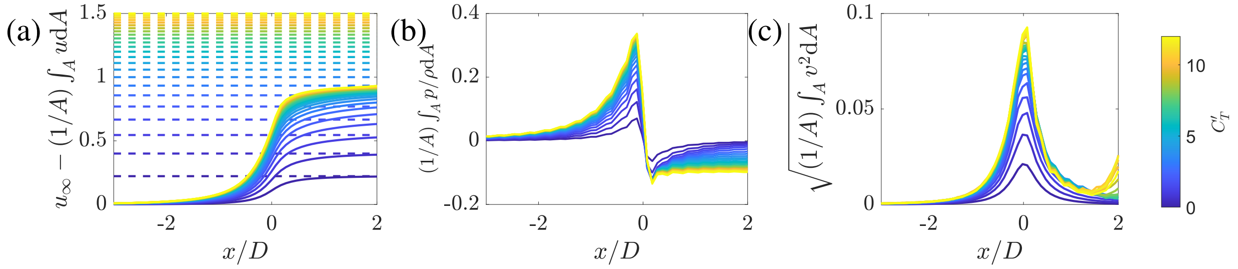

Figure S10: The streamtube averaged values of (a) streamwise velocity deficit normalized by the freestream wind speed, (b) pressure, and (c) root-mean-square of the velocity normalized by the freestream wind speed. (a) Dashed lines are classical momentum modeling predictions neglecting the wake pressure deficit. Increasingly light colors correspond to increasing C_T', from 0.5 to 12.

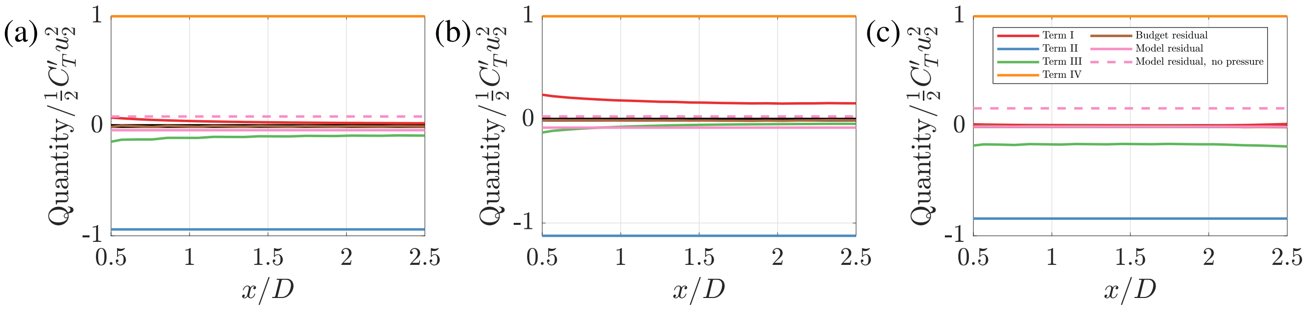

Figure S11: Streamtube integrated energy conservation from Eq. (89), for (a) $C_T' = 4$ and $\gamma = 0°$, (b) $C_T' = 4$ and $\gamma = 40°$, and (c) $C_T' = 12$ and $\gamma = 0°$. In addition to the individual budget terms, the residual is shown. Model approximations of the budget are also shown, where each term is approximated by a single averaged value that is characteristic of the 'near-wake.' The model approximation residuals are shown with and without the pressure contribution, to demonstrate the influence of neglecting the wake pressure p_4 in energy conservation.

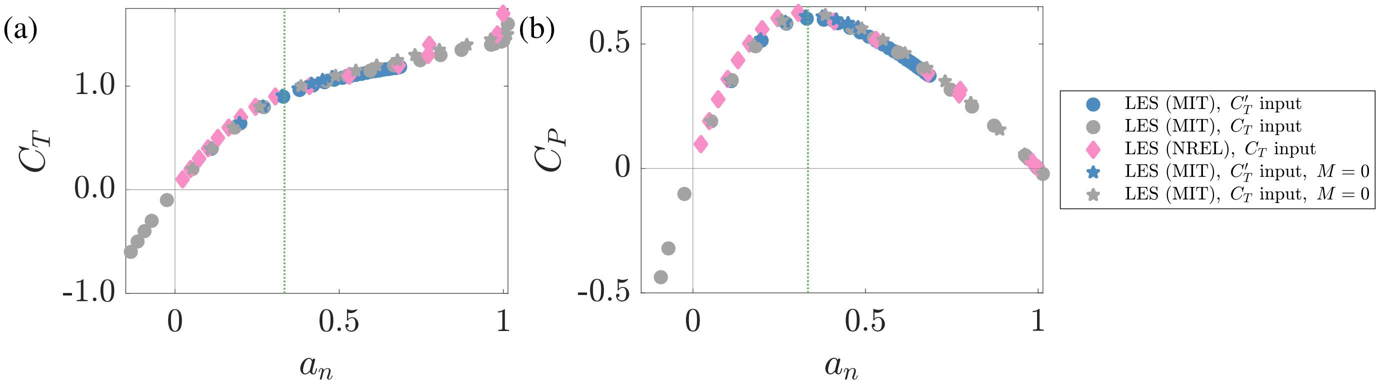

Figure S12: (a) Coefficient of thrust C_T and (b) Coefficient of power C_P as a function of the rotor-normal induction factor a_n. Results are shown for different LES codes and different numerical implementations of the actuator disk model forcing. The Betz limit of $a_n = 1/3$ ($C_T' = 2$) and $C_P = 16/27$ is also shown by the dashed green line.

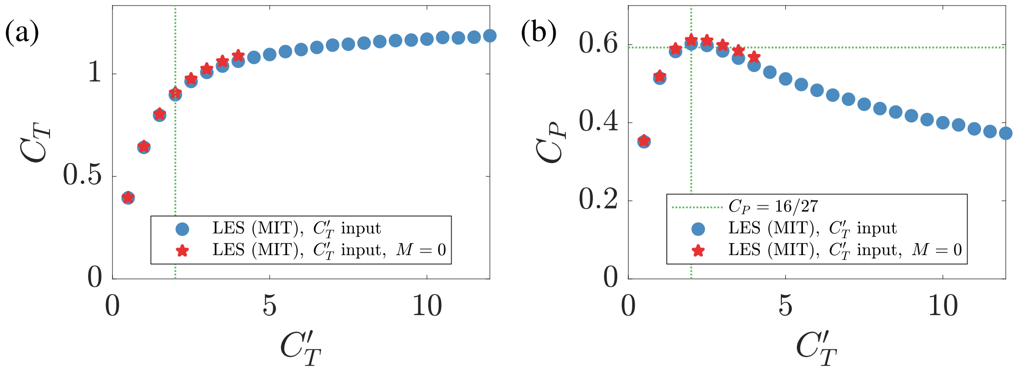

Figure S13: (a) Coefficient of thrust C_T and (b) Coefficient of power C_P as a function of the local thrust coefficient C'_T. Results are shown for different LES codes and different numerical implementations of the actuator disk model forcing. The Betz limit of $a_n = 1/3$ ($C'_T = 2$) and $C_P = 16/27$ is also shown by the dashed green line.

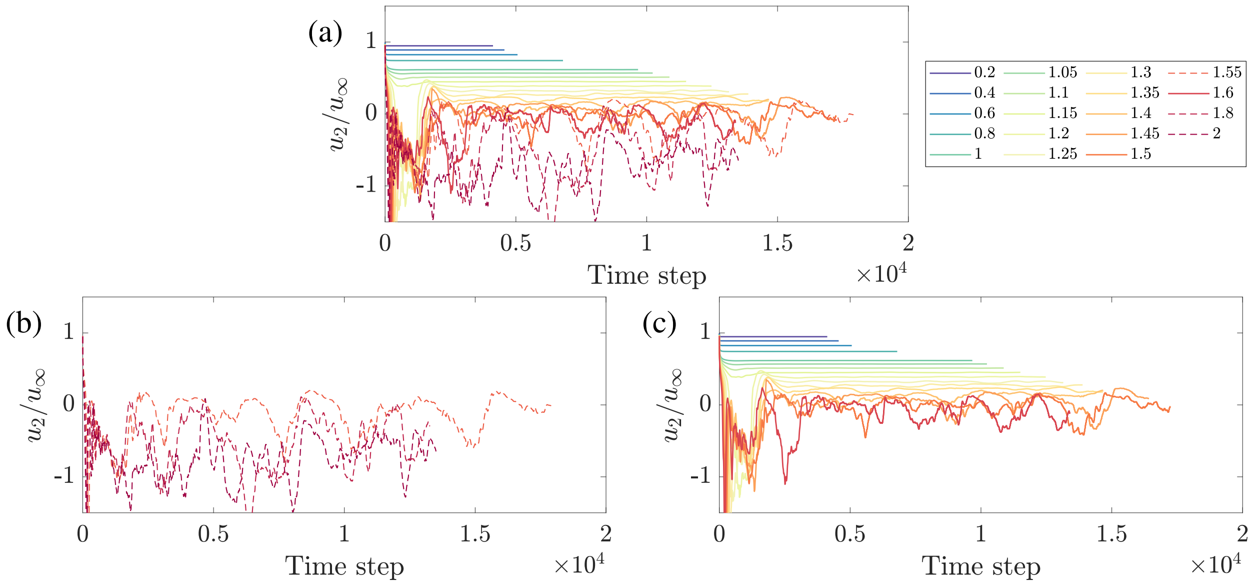

Figure S14: Time series of the disk velocity u_2 normalized by the freestream wind speed u_∞ for different input C_T values for the actuator disk model in large eddy simulation. (a) All C_T values simulated. (b) C_T values where the disk velocity standard deviation $\sqrt{\overline{u_2'^2}} > 0.15u_\infty$. (c) C_T values where the disk velocity standard deviation $\sqrt{\overline{u_2'^2}} < 0.15u_\infty$. In all figures, values of C_T with $\sqrt{\overline{u_2'^2}} > 0.15u_\infty$ are shown with dashed lines, and $\sqrt{\overline{u_2'^2}} < 0.15u_\infty$ are shown with solid lines. Only results from the C_T values in subfigure (c) are included in the main text. The legend indicates the C_T values for each line.

Supplementary References

1. K. S. Heck, H. M. Johlas, M. F. Howland, *Journal of Fluid Mechanics* **959**, A9 (2023).
2. G. A. Van Kuik, *Wind Energy: An International Journal for Progress and Applications in Wind Power Conversion Technology* **10**, 289 (2007).
3. L. Su, *Fundamentals of incompressible fluid mechanics I. Inviscid flows* (Stanford University, 2013).
4. B. R. Munson, A. P. Rothmayer, T. H. Okiishi, *Fundamentals of fluid mechanics* (Wiley Global Education, 2012).
5. C. R. Shapiro, D. F. Gayme, C. Meneveau, *J. Fluid Mech.* **841**, R1 (2018).
6. L. M. Milne-Thomson, *Theoretical Aerodynamics* (Courier Corporation, 1973).
7. J. H.-w. Lee, V. H. Chu, *Turbulent jets and plumes: a Lagrangian approach*, vol. 1 (Springer Science & Business Media, 2003).
8. M. Bastankhah, F. Porté-Agel, *J. Fluid Mech.* **806**, 506 (2016).
9. H. A. Madsen, *Wind Energy Science* **8**, 1853 (2023).
10. T. von Kármán, J. M. Burgers, *Aerodynamic Theory: General Aerodynamic Theory-Perfect Fluids*, vol. II (Springer, Berlin, Heidelberg, 1935).
11. J. Kim, P. Moin, *Journal of computational physics* **59**, 308 (1985).
12. A. Dicholkar, F. Zahle, N. N. Sørensen, *Journal of Wind Engineering and Industrial Aerodynamics* **220**, 104863 (2022).
13. P. C. Chu, J. H. Lee, V. H. Chu, *Journal of Hydraulic Engineering* **125**, 193 (1999).

14. M. Albertson, Y. Dai, R. A. Jensen, H. Rouse, *Transactions of the American Society of Civil Engineers* **115**, 639 (1950).

15. N. E. Kotsovinos, *Journal of Fluid Mechanics* **77**, 305 (1976).

16. D. R. Miller, E. W. Comings, *Journal of Fluid Mechanics* **3**, 1 (1957).

17. L. Bradbury, *Journal of Fluid Mechanics* **23**, 31 (1965).

18. M. Bastankhah, F. Porté-Agel, *Renewable Energy* **70**, 116 (2014).

19. K. Steiros, M. Hultmark, *Journal of Fluid Mechanics* **853**, R3 (2018).

20. G. Taylor, *Aeronautical Research Council, Reports and Memoranda* **2236**, 159 (1944).

21. J.-K. Koo, D. F. James, *Journal of Fluid Mechanics* **60**, 513 (1973).

22. F. O'Neill, *Ocean engineering* **33**, 1884 (2006).

23. N. Bempedelis, K. Steiros, *Physical Review Fluids* **7**, 034605 (2022).

24. H. Glauert, *et al.*, *A general theory of the autogyro*, vol. 1111 (HM Stationery Office, 1926).

25. H. Glauert, *Aerodynamic theory* (Springer, 1935), pp. 169–360.

26. M. L. Buhl Jr, New empirical relationship between thrust coefficient and induction factor for the turbulent windmill state, *Tech. rep.*, National Renewable Energy Lab (NREL), Golden, CO (United States) (2005).

27. R. E. Wilson, *Wind Turbine Technology: Fundamental Concepts in Wind Turbine Engineering, Second Edition*, D. A. Spera, ed. (ASME Press, 2009), pp. 215–282.

28. R. E. Wilson, P. B. S. Lissaman, Applied aerodynamics of wind power machines, *Tech. Rep. PB-238595*, Oregon State Univ., Corvallis (USA) (1974).

29. T. Burton, N. Jenkins, D. Sharpe, E. Bossanyi, *Wind energy handbook* (John Wiley & Sons, 2011).

30. H. A. Madsen, T. J. Larsen, G. R. Pirrung, A. Li, F. Zahle, *Wind Energy Science* **5**, 1 (2020).

31. E. Branlard, *Wind turbine aerodynamics and vorticity-based methods: Fundamentals and recent applications* (Springer, 2017).

32. J. Sørensen, W. Shen, X. Munduate, *Wind Energy: An International Journal for Progress and Applications in Wind Power Conversion Technology* **1**, 73 (1998).

33. E. Gaertner, *et al.*, IEA wind TCP task 37: Definition of the IEA 15-megawatt offshore reference wind turbine, *Tech. rep.*, National Renewable Energy Lab.(NREL), Golden, CO (United States) (2020).

34. C. Cossu, *Wind Energy Science* **6**, 377 (2021).

35. M. A. Sprague, S. Ananthan, G. Vijayakumar, M. Robinson, *Journal of Physics: Conference Series* (IOP Publishing, 2020), vol. 1452, p. 012071.

36. E. Branlard, *et al.*, *Journal of Physics: Conference Series* (IOP Publishing, 2024).

37. M. F. Howland, J. Bossuyt, L. A. Martínez-Tossas, J. Meyers, C. Meneveau, *J. Renew. Sustain. Energy* **8**, 043301 (2016).

38. C. R. Shapiro, D. F. Gayme, C. Meneveau, *Wind Energy* **22**, 1414 (2019).

39. L. A. Martínez-Tossas, *et al.*, *Wind Energy* **25**, 605 (2022).

40. W. Munters, J. Meyers, *Philosophical Transactions of the Royal Society A: Mathematical, Physical and Engineering Sciences* **375**, 20160100 (2017).

41. F. Stoddard, *Wind Technology Journal* **1**, 3 (1978).

42. F. Nicoud, H. B. Toda, O. Cabrit, S. Bose, J. Lee, *Phys. Fluids* **23**, 085106 (2011).

43. L. Prandtl, Applications of modern hydrodynamics to aeronautics, *Tech. Rep. TR-116*, National Advisory Committee for Aeronautics (1923).
